# Correction to: Antifungal activity of silver nanoparticles on fungal isolates from patients of suspected mucormycosis

**DOI:** 10.1007/s10123-022-00285-2

**Published:** 2022-11-03

**Authors:** Kuhu Chatterjee, Juhi Taneja, Shilpa Khullar, Anil Kumar Pandey

**Affiliations:** 1 Department of Microbiology, ESIC Medical College and Hospital, NH‑3 NIT, Faridabad, Haryana India; 2 Department of Physiology, ESIC Medical College and Hospital, NH‑3 NIT, Faridabad, Haryana India

**Correction to** **:** **International Microbiology**

**https://doi.org/10.1007/s10123-022-00280-7**

The article Antifungal activity of silver nanoparticles on fungal isolates from patients of suspected mucormycosis, written by Kuhu Chatterjee, Juhi Taneja, Shilpa Khullar, and Anil Kumar Pandey, was originally published online on the publisher’s internet portal on October 17, 2022 with Open Access under a “Creative Commons Attribution (CC BY) license 4.0”. With the author’s/authors' decision to cancel Open Access, the copyright of the article changed on October 17, 2022 to © The Author(s), under exclusive licence to Springer Nature Switzerland AG 2022 with all rights reserved.

The original article has been corrected.

